# HOW TO STEADI YOUR PATIENTS WITH THE COORDINATED CARE PLAN TO PREVENT OLDER ADULT FALLS AND EVALUATION GUIDE

**DOI:** 10.1093/geroni/igz038.3152

**Published:** 2019-11-08

**Authors:** Janice A Mark

**Affiliations:** Centers for Disease Control and Prevention, Atlanta, Georgia, United States

## Abstract

Falls are common, costly, and the leading cause of fatal and nonfatal injuries for older Americans. Reports show that fall death rates are increasing. Healthcare providers play an important role in fall prevention but few talk to their patients about falls. This lack of communication demonstrates the need for more physician-initiated fall prevention. The Centers for Disease Control and Prevention (CDC) created the Stopping Elderly Accidents, Deaths, and Injuries (STEADI) initiative to help providers talk to their patients about falls. Specifically, CDC’s new STEADI-based fall prevention program, the Coordinated Care Plan to Prevent Older Adult Falls (CCP) and Evaluation Guide for Older Adult Clinical Fall Prevention Programs can assist healthcare providers in integrating and evaluating new fall prevention programs that screen older adults for fall risk, assess patients’ modifiable fall risk factors, and implement evidenced-based fall prevention interventions (e.g., medication management, physical therapy). The CCP offers guidance for incorporating a STEADI-based fall prevention program including how to engage leadership, integrate with existing clinic workflow and electronic health records, and strategies on how to obtain reimbursement for fall prevention. The Evaluation Plan offers details on how to engage stakeholders, collect data, interpret findings and how to share results for maximum impact. Both documents were based on lessons learned from successful implementation of STEADI-based programs in primary care. A STEADI-based program in New York found fewer fall-related hospitalizations among at-risk patients who received a fall prevention care plan compared to at-risk patients who did not receive a care plan.

